# Adaptability and Stability Analyses of Root Yield and Micronutrient Concentrations in Sweetpotato (*Ipomoea batatas* [L.] Lam.) Genotypes

**DOI:** 10.1002/pei3.70155

**Published:** 2026-06-01

**Authors:** Nomusa Chizhande, Julia Sibiya, Edmore Gasura, Bruce Mutari, Isack Mathew, Miriam Chibvongodze

**Affiliations:** ^1^ School of Agricultural, Earth and Environmental Sciences University of KwaZulu‐Natal Pietermaritzburg South Africa; ^2^ Department of Research and Specialist Services, Crops Research Division Harare Zimbabwe; ^3^ Vision for Adapted Crops and Soils (VACS) Capacity, SAS Program International Maize and Wheat Improvement Center, (CIMMYT) Nairobi Kenya; ^4^ Department of Plant Production Sciences and Technologies University of Zimbabwe Harare Zimbabwe; ^5^ Alliance for a Green Revolution in Africa Nairobi Kenya; ^6^ Department of Plant & Soil Sciences University of Venda, FESA Thohoyandou South Africa

**Keywords:** AMMI, Fe, GGE, multi‐environment, root tuber yield, stability analysis, Zn

## Abstract

High yielding and micronutrient dense sweetpotato cultivars could contribute to alleviating micronutrient deficiency, which is a prevalent problem in developing countries. Developing these improved cultivars involves selection across diverse environments, which requires an understanding of genotype × environment interaction (GEI). This study evaluated root yield (RY), β‐carotene, iron (Fe), and zinc (Zn) concentrations in 22 sweetpotato genotypes across different locations to identify superior genotypes for broad and specific adaptation. Trials were conducted at four different locations across two seasons in a randomized block design with two replications. The data were subjected to the genotype and genotype by environment (GGE) biplot analyses. Significant (*p* < 0.05) genotype × environment interactions were reported for fresh root yield, β‐carotene, Fe and Zn content. There was wide genotypic variation in RY and micronutrient contents. The most desirable genotypes were G3, G19 and G16 with higher than 7.5 t/ha of RY and G19, G12 and G16 that had high β‐carotene. These genotypes were selected for breeding for improved cultivars. Overall, seven genotypes G3, G19, G16, G22, G4, G5 and G2 with high and stable RY and high micro‐nutrient concentrations were selected for developing breeding populations. These genotypes are recommended for sweetpotato breeding programme in Zimbabwe and similar environments.

## 1 Introduction

Sweetpotato ( *Ipomoea batatas* [l.] lam) is widely grown for its tubers that are high in starch, and micronutrient content. It is highly nutritious with starch being the main nutrient at 20%–26%, while protein (2%–3%), vitamins (vitamin A: approximately 28,355 IU per 100 g, vitamin C: about 2.4 mg per 100 g, vitamin B6: around 0.209 mg per 100 g, vitamin E: roughly 0.26 mg per 100 g) and minerals such as potassium (542 mg/g), phosphorous (40–60 mg/g) and calcium (30 mg/g) are also available in the tuber (Nandutu and Howell 2009). Its high nutritional content could be harnessed to address dietary deficiencies in micronutrient such as iron (Fe) and zinc (Zn) prevalent in developing countries (Bouis and Saltzman 2017; Gregory et al. 2017; Kihara et al. 2020). However, there are very few sweetpotato varieties that combine high yield potential with high micronutrient contents. It is imperative to develop suitable varieties with high yield potential and high concentrations of micronutrients such as β‐carotene, Fe and Zn. While sweetpotato contains a broad spectrum of nutrients, this study focuses specifically on β‐carotene, Fe, and Zn due to their major public health importance. Vitamin A deficiency is a leading cause of preventable blindness, while Fe and Zn deficiencies are associated with anemia, impaired cognitive development, and weakened immune systems. These nutrients are also key targets in global biofortification programs. However, other nutrients such as vitamin C, potassium and dietary fiber are also important and should be explored in future studies to provide a more comprehensive nutritional evaluation.

Breeding for improved cultivars with desirable characteristics requires evaluation of genotypes across target environments. However, genotype × environment interactions (GEI) effects affect agronomic performance, which confounds the selection process and reduces breeding efficiency (Adebola et al. 2013; Begna 2020; Merga 2022). Genotype by environment interaction (GEI) reduces the correlation between genotype and phenotypic expression, and ultimately reduces potential genetic gain (Van Oosterom et al. 1993). Differentials in genotype performance across environments have been attributed to GEI and can be used to determine whether a genotype exhibits wide or specific adaptation to the environments (Yan 2001; Abidin et al. 2005). A genotype that exhibits wide adaptation has stable yield across multiple environments (Mwanga and Ssemakula 2011; Brown et al. 2020). Conversely, a genotype with specific adaptation is particularly high yielding in a narrow range of environments (Brown et al. 2020). It is imperative to evaluate genotypes in multiple environments to identify superior and adapted genotypes for developing breeding populations and improved cultivars.

A wide range of techniques have been used to analyze the adaptability and stability of genotypes (Efroni et al. 2008; Metzger et al. 2009; Pereira et al. 2009). Genotype and genotype by environment (GGE) biplot (Yan 2001), and additive main effects and multiplicative interaction (AMMI) analyses (Gauch and Zobel 1997) are widely used in multi‐environment trials (MET). The GGE partitions the variation into genotype and GxE components but do not account for the environment. In contrast, the AMMI decomposes the variation into genotype and environment main effects and the GxE multiplicative component. The GGE approach is useful for identifying superior genotypes and discriminating environments while the AMMI is more useful for prediction and modeling (Yan and Kang 2003; Gauch 2006). Combined, these two approaches provide robust methods for the breeder to identify and select superior genotypes and important environments while offering a method to predict untested genotypes or environments. The main difference between the two methods being the inclusion of the genotype main effect as a multiplicative effect and not as an additive main effect in AMMI biplots (Kang et al. 2003). In addition to identifying stable genotypes, GGE also determines the most representative and discriminating test environments for cultivar evaluation. The objectives of this study were to evaluate genotype performance in root yield production and β‐carotene content, Fe and Zn contents across different environments and determine the genotype × environment interaction (GEI) effects affecting trait expression.

## 2 Materials and Methods

### 2.1 Plant Materials

The study investigated 22 sweetpotato genotypes that were sourced from the International Potato Centre (ICP), in Mozambique, and Crop Breeding Institute in Zimbabwe (Table 1). The CIP materials are introductions to Zimbabwe and have not been tested for agronomic performance and nutritional content. Their inclusion will contribute to the available genetic diversity for sweetpotato breeding. The genotypes from Zimbabwe are landraces that are commonly grown and adapted to local conditions. The landraces have been evaluated for agronomic performance, but their nutritional value is relatively less documented.

**TABLE 1:** Description of the genotypes used in the study.

Code	Genotype	Source^a^	Flesh color	Skin color	Type
G1	Red Jewel	CBI‐Zimbabwe	Cream	Cream	Landrace
G2	Germany 2	CBI‐Zimbabwe	White	Pink	Landrace
G3	Dube	CBI‐Zimbabwe	Cream	Cream	Landrace
G4	Cordner	CBI‐Zimbabwe	Orange	Cream	Landrace
G5	Chigondo	CBI‐Zimbabwe	Cream	Cream	Landrace
G6	Harare	CBI‐Zimbabwe	Cream	Cream	Landrace
G7	Fost	CBI‐Zimbabwe	White	Pink	Landrace
G8	Shirikadzi	CBI‐Zimbabwe	White	Pink	Landrace
G9	Kau 4	CBI‐Zimbabwe	Cream	Cream	Landrace
G10	Irene	CBI‐Zimbabwe	Cream	Cream	Landrace
G11	Nyekete	CBI‐Zimbabwe	Cream	Cream	Landrace
G12	Beauregard	CBI‐Zimbabwe	Orange	Orange	Released
G13	O‐101	CIP‐Mozambique	Orange	Orange	Clone
G14	O‐102	CIP‐Mozambique	Orange	Orange	Clone
G15	O‐103	CIP‐Mozambique	Orange	Orange	Clone
G16	O‐104	CIP‐Mozambique	Orange	Orange	Clone
G17	O‐105	CIP‐Mozambique	Orange	Orange	Clone
G18	O‐106	CIP‐Mozambique	Orange	Pink	Clone
G19	P‐201	CIP‐Mozambique	Orange	Orange	Clone
G20	P‐202	CIP‐Mozambique	Orange	Cream	Clone
G21	P‐203	CIP‐Mozambique	Purple white	Purple	Clone
G22	Chingovha	CBI‐Zimbabwe	Cream	Cream	Released

Abbreviation: CBI, crop breeding institute.

^a^ CIP International Potato Centre in Mozambique.

### 2.2 Experimental Sites

The experiments were established in four locations over two seasons (2021/22 and 2022/23) giving a total of eight environments (season × location). The locations included Makoholi Research Station, which is in the lowveld region of Zimbabwe and represented a low potential environment. The other three locations were the Harare Research Station, the Gwebi Variety Testing Center, and the Horticulture Research Center, all in Zimbabwe's highveld region and represented high potential environments. The climatic conditions, soil types, micronutrient profiles, and agro‐ecological characteristics of the locations were diverse (Table 2). Soil samples for site characterization were collected prior to planting from the topsoil (0 to 20 cm horizon) using a composite sampling approach. At each site, multiple subsamples were collected randomly across the field, mixed thoroughly, and a representative composite sample was analyzed for physicochemical properties.

**TABLE 2:** Description of the experimental locations used in the study.

Parameter	HRS	GVTC	HRC	MKHL
Latitude	18.11S	17.69S	18.11S	19.50S
Longitude	31E	30E	31E	30E
Altitude (m.a.s.l)	1506	1449	1630	1204
pH (calcium chloride)	5.4	6.1	5.4	5.7
OM (%)	2.1	2.0	0.7	2.3
N (ppm)	19	18	13	22
P (ppm)	73	67	70	77
Ca (mg/100 g)	9.0	10.7	8.5	9.1
Mg (mg/100 g)	5.6	6.3	6.7	6.0
K (mg/100 g)	0.24	0.38	0.28	0.35
Zn (ppm)	2.40	1.46	0.57	2.19
Fe (ppm)	13.13	10.27	6.11	7.05
Soil type	Clay	Clay loam	Sandy‐loam	Sandy‐clay

Abbreviations: GVTC, Gwebi Variety Testing Center; HRC, Horticulture Research Centre; HRS, Harare Research Station; masl, meters above sea level; mg/100 g, milligram equivalents per 100 g; MKHL, Makoholi Experiment Station; mm, millimeters; OM, organic matter content; ppm, parts per million.

### 2.3 Experimental Design, Field Management

The trials were established under rain‐fed conditions and laid out in a randomized block design with two replications at each of the locations. The use of two replicates was guided by logistical constraints typical of multi‐location trials and is supported by the use of robust statistical models (AMMI and GGE), which effectively account for environmental variability and improve the precision of genotype evaluation. Previous studies have demonstrated that even with limited replication, reliable genotype ranking can be achieved when environments are sufficiently diverse. Each plot consisted of four 6 m long rows, with inter‐row and intra‐row spacing of 0.90 m and 0.30 m, respectively. Weeding was carried out manually when required and standard agronomic practices for sweetpotato were applied.

### 2.4 Data Collection

Harvesting was done at physiological maturity, which was approximately 135 days after planting. Two middle rows were sampled for harvesting the root tubers. The root tubers were then weighed for root fresh weight and nutritional quality testing using a technique developed by Cole et al. (2014). Upon harvesting, the storage roots were separated into two categories, mainly productive and unproductive before weighing. The number of productive fresh storage roots was counted and recorded on a per plot basis.

To determine the nutritional content, three tubers of varying sizes were selected per plot. The selected storage roots were properly cleaned with tap water and then rinsed with distilled water. Thereafter, they were peeled and chopped into small pieces. The chopped tubers were then weighed to 100 g composite samples and placed in a glass petri dish. The samples were oven dried at 70°C before milling. The samples were then ground in a stainless cutting mill until they could pass a 1 mm sieve. They were then kept in plastic containers with tight‐fitting covers until their mineral composition could be ascertained.

Micronutrient content analysis was carried out at the Department of Research and Specialist Services (DR&SS) in Harare, Zimbabwe, at the food laboratory of the Chemistry and Soils Research Institute. Using the atomic absorption spectrophotometer model, 50 g of milled dry sweetpotato root samples were evaluated for Fe and Zn contents following a protocol described in Elango et al. (2021). The wavelengths used were 2483A for the iron and 2133A for zinc. The β‐carotene content was determined using spectrophotometry according to the protocol developed by van Jaarsveld et al. (2006) and the results were expressed as μg per 100 g.

### 2.5 Data Analysis

#### 2.5.1 Analysis of Variance

A combined analysis of variance across environments was done using GenStat 18th edition (Payne et al. 2018) statistical package. The following statistical model was used:

$$Y_{\text{ijklm}}=\mu +G_{i}+L_{j}+R_{l\left(\mathrm{jk}\right)}+B_{m\left(\mathrm{jkl}\right)}+{\mathrm{GL}}_{\mathrm{ij}}+{\mathrm{GY}}_{\mathrm{ik}}+{\mathrm{LY}}_{\mathrm{jk}}+{\mathrm{GLY}}_{\mathrm{ijk}}+e_{\text{ijklm}}$$

where *Y*_*ijklm*_ = response of the ith genotype in *j*th location and *l*th replication within year and *m*th block within location, *μ* = grand mean, *G*_*i*_ = random effect of the ith genotype, *L*_*j*_ = fixed effect of the _j_th location, Y_k_ = fixed effect of the year *k*, *R*_l(jk)_ = fixed effect of the replicate *l* nested within location *j* in year *k*, *B*_*m*(*jkl*)_ = fixed effect of the block *m* nested within location *j* in year *k* and replicate *l*, *GL*_*ij*_ = random effect of the interaction between genotype *i* and location *j*, *GY*_*ik*_ = random effect of the interaction between genotype *i* and year *k*, *LY*_*jk*_ = random effect of the interaction between location *j* and year *k*, *GLY*_*ijk*_ = random effect of genotype by location by year interaction, and *e*_*ijlkm*_ = random error.

The AMMI stability value (ASV) was calculated to quantify and rank genotypes (Rezene et al., 2014). This was carried out using a formula provided by Purchase et al. (2000);

$$\text{AMMI Stability Value}\;\left(\mathrm{ASV}\right)=\sqrt{\left[{\left(\frac{\text{SSIPCA}1}{\text{SSIPCA}2}\left(\text{IPCA}1\right)\right)}^{2}+{\left[\text{IPCA}2\right]}^{2}\right]}$$

where $\frac{\text{SSIPCA}1}{\text{SSICPA}2}$ represents the weighted value assigned to the first interaction principal component score due to its high contributions in the GXE model, SSIPCA1 and SSIPCA2 are the sum of squares for IPCA1 and IPCA2, respectively, IPCA1 and IPCA2 are the first and second IPCA scores for 155 each genotype. The larger the ASV value the more specifically adapted the genotype to a certain environment and the smaller ASV indicates a more stable genotype across environments (Purchase et al. 2000; Geravandi et al. 2011; Thiyagu et al. 2012).

The model for a GGE biplot (Yan 2001; Yang et al. 2007) based on singular value decomposition (SVD) of t principal components is:

$${Ȳ}_{\mathrm{ij}}-\mu_{i}-\beta_{j}=\sum_{k=1}^{t}\lambda_{k}\alpha_{\mathrm{ik}}\gamma_{\mathrm{jk}}=\varepsilon_{\mathrm{ij}}$$

where ${Ȳ}_{\mathrm{ij}}$ is the performance of genotype *i* in environment *j*, *μ* is the grand mean, *β*_*j*_ is the main effect of environment *j*, *k* is the number of principal components (PC); *λ*_*k*_ is singular value of the *k*th PC; and *α*_*ik*_ and *γ*_*jk*_ are the scores of *i*th genotype and *j*th environment, respectively for PC_*k*_; *ε*_*ij*_ is the residual associated with genotype *i* in environment *j*.

## 3 Results

### 3.1 Combined Analysis of Variance

The combined analysis of variance (ANOVA) for root yield, β‐carotene, iron, and zinc content across 22 sweetpotato genotypes evaluated in eight environments is summarized in Table 3. Statistically significant differences (*p* < 0.001) were detected among genotypes for root yield, iron, and zinc, while differences in β‐carotene content were significant at the *p* < 0.05 level. The effects of incomplete block structures within environments [Block (Env*Rep)] were also highly significant (*p* < 0.001), indicating substantial variation due to experimental design factors. Significant GEI effects (*p* < 0.001) were observed for β‐carotene, iron, and zinc. These findings indicate that the expression of β‐carotene, iron, and zinc in sweetpotato genotypes was significantly influenced by environmental conditions and their interaction with genotypic performance.

**TABLE 3:** Combined analysis of variance for root yield, β‐carotene, iron, and zinc concentrations of 22 sweetpotato genotypes evaluated across four locations in Zimbabwe.

Source of variation	Df	Root yield		β‐carotene		Iron		Zinc	
		SS	MS	SS	MS	SS	MS	SS	MS
Env	3	165482569	55160856***	17.895	5.965*	116237.7	38745.9***	4489.489	1496.496***
Rep(Env)	4	6804919	1701230	0.001	0	1360.8	340.2***	4.045	1.011
Block(Env*Rep)	8	58256777	7282097**	2089.015	261.127***	30047.1	3755.9***	106.449	13.306**
Gen	21	633907400	30186067***	5235.72	249.32***	67267.1	3203.2***	857.666	40.841***
Gen*Env	60	95982115	1599702	219.548	3.659***	98476.9	1641.3***	2867.215	47.787***
Residual	255	719197770	2820383	509.966	2	179146.2	702.5	1212.034	4.753

*Note:* *, **, ***indicate significance at *p* ≤ 0.05, *p* ≤ 0.01 and *p* ≤ 0.001, respectively.

Abbreviations: Block(Env*Rep), incomplete block within an environment; df, degrees of freedom; Env, environment; Gen*Env, genotype by environment interaction; Gen, genotype; MS, mean square error; ppm, parts per million; Rep(Env), Replications nested in environments; SS, mean sum of squares.

### 3.2 Genotype Mean Performance for Root Yield and Nutrient Content

The root yield, β‐carotene, Fe and Zn mean performance of the 22 sweetpotato genotypes are presented in Table 4. The mean β‐carotene content of the genotypes ranged between 0.0 and 13.47 mg/100 g. Genotype G17 expressed the highest β‐carotene content of 13.47 mg/100 g, whilst eight genotypes, G5, G22, G3, G7, G2, G6, G10, G9, G11, G21, G1 and G8, had no β‐carotene. Genotype G15 had the lowest Fe content of 50.12 ppm while the highest Fe content was recorded for genotype G18 with 108.06 ppm. Genotype G12 expressed the highest Zn content of 20.07 ppm and genotype G15 recorded the lowest Zn content. Mean root yield ranged from 1.02 t/ha (G1) to 8.38 t/ha (G3), with a grand mean RY of 6.18 t/ha, while 59% of the genotypes had mean root yield that was greater than the average. Although G3 and G19 were the highest yielding genotypes, these two were not among the top performers with respect to root β‐carotene content, Fe and Zn concentrations (Table 4).

**TABLE 4:** Mean performances for root yield, beta carotene, iron and zinc across eight environments and two growing seasons.

Genotype	Yield (kg/ha)	Rank	Genotype	Beta carotene (mg/100 g)	Rank	Genotype	Iron (ppm)	Rank	Genotype	Zinc (ppm)	Rank
G3	8382^a^	1	G17	13.477^a^	1	G18	108.06^a^	1	G12	20.37^a^	1
G19	7689^ab^	2	G14	12.446^b^	2	G13	96.44^ab^	2	G20	19.69^b^	2
G16	7458^abc^	3	G20	11.811^c^	3	G14	86.25^bc^	3	G16	19.19^c^	3
G22	7378^a‐d^	4	G19	7.661^d^	4	G8	86.12^bc^	4	G14	18.5^d^	4
G2	7179^b‐e^	5	G12	7.23^e^	5	G17	84.5^bc^	5	G8	18.19^d^	5
G12	6815^b‐e^	6	G16	6.41^f^	6	G6	82.5^cd^	6	G17	17.37^e^	6
G4	6599^b‐e^	7	G18	6.001^g^	7	G12	81.0^cd^	7	G19	17.25^ef^	7
G17	6421^cde^	8	G15	5.66^h^	8	G20	80.37^cd^	8	G21	17.00^efg^	8
G18	6385^c‐f^	9	G4	3.227^i^	9	G22	77.87^cd^	9	G4	16.81^fgh^	9
G5	6383^c‐f^	10	G13	0.112^j^	10	G16	77.19^cd^	10	G7	16.81^fgh^	10
G20	6361^c‐f^	11	G9	0.03^k^	11	G3	74.5^cde^	11	G10	16.5^ghi^	11
G14	6212^d‐g^	12	G2	0.016^l^	12	G10	70.0^def^	12	G6	16.56^ghi^	12
G11	6200^d‐g^	13	G6	0.015^l^	13	G1	69.06^def^	13	G22	16.44^hi^	13
G7	6173^d‐h^	14	G10	0.015^l^	14	G7	68.06^def^	14	G1	16.37^hi^	14
G15	6146^d‐h^	15	G5	0.009^m^	15	G9	68.00^def^	15	G18	16.31^hi^	15
G9	6040^e‐h^	16	G7	0.005^mn^	16	G2	62.69^efg^	16	G13	16.19^ij^	16
G8	6038^e‐h^	17	G22	0.004^mn^	17	G21	58.75^fg^	17	G5	15.75^jk^	17
G10	5918^e‐h^	18	G8	0.004^mn^	18	G19	58.06^fg^	18	G3	15.44^k^	18
G21	5153^fgh^	19	G3	0.002^n^	19	G5	55.81^fg^	19	G11	15.31^k^	19
G13	5066^gh^	20	G11	0^n^	20	G4	55.56^fg^	20	G2	14.69^l^	20
G6	4969^h^	21	G21	0^n^	21	G11	51.69^g^	21	G9	14.62^l^	21
G1	1017^i^	22	G1	0^n^	22	G15	50.12^g^	22	G15	13.5^m^	22
Grand Mean	6181			3.37			72.85			16.767	
Min	969			0			50.12			13.5	
Max	8382			13.47			108.06			20.37	
CV	3.6			21.3			8.3			7.5	
LSD	132.65			0.04			6.47			3.44	

*Note:* Means with different letters (a‐i) within the same column are significantly different.

### 3.3 Stability Analysis Using Yield Stability Index and AMMI Stability Value

The ASV of the genotypes across environments ranged from 1.36 for genotype G3 to 13.55 for genotype G1 (Table 5). Based on ASV, the most stable genotypes were G3, G19, G16, G22, G4, G5, and G2 that exhibited low ASV while genotypes such as G1, G6, G11, G10, G15, and G13 with high ASV were the least stable. The simultaneous selection of the genotypes for root yield and stability performances showed G3 as the most stable and adapted genotype with high YSI, followed by G19, G16, G22, G4, G2, G5, G20, G17, G8, and G1. Genotypes G6, G21, G10, G11, and G15 were the least stable genotypes as they exhibited low YSI values. In addition, both statistical models identified G1, G6, and G21 among the most unstable genotypes in terms of root yield.

**TABLE 5:** Ranking of the 22 genotypes based on yield stability index (YSI) and AMMI stability AMMI analysis value (ASV).

Genotype	YSI	ASV	Rank
G3	1.483	1.364	1
G19	1.4135	4.404	2
G16	1.344	4.581	3
G22	1.2744	4.994	4
G4	1.2049	5.207	5
G2	1.1354	5.571	6
G5	1.0659	5.884	7
G20	0.9963	6.248	8
G17	0.9268	6.42	9
G8	0.8573	6.459	10
G7	0.7878	7.179	11
G14	0.7182	7.703	12
G18	0.6487	7.752	13
G9	0.5792	7.862	14
G12	0.5097	8.762	15
G13	0.4401	9.521	16
G11	0.3706	9.604	17
G15	0.3011	9.667	18
G10	0.2316	9.698	19
G21	0.162	9.857	20
G6	0.0925	9.972	21
G1	0.023	13.554	22

### 3.4 Genotype Stability and GE Interaction

**FIGURE 1:**
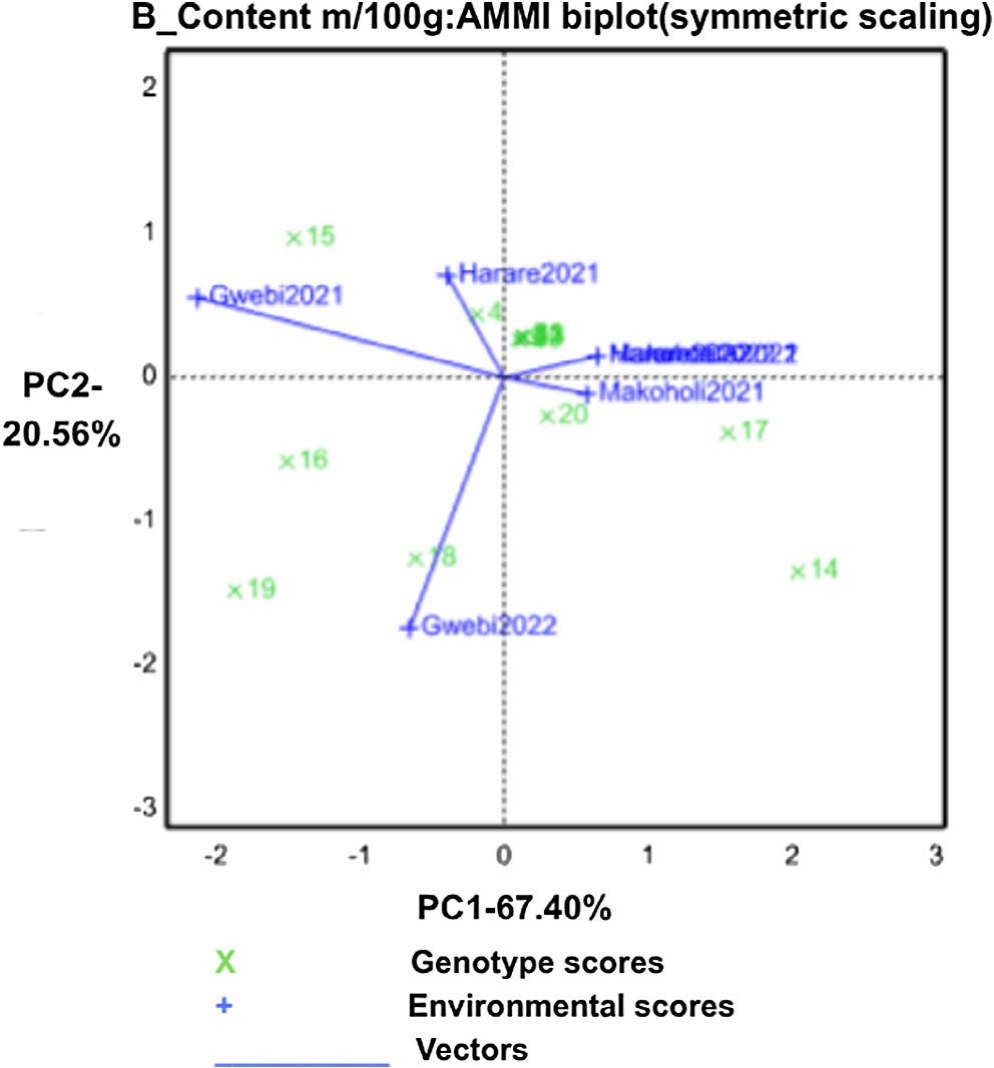
GGE‐biplot showing interrelationship among environments and discriminating ability of the environments for β‐carotene.

The GGE biplot (Figure 1) illustrates relationships among environments and their ability to discriminate genotypes for β‐carotene concentration. PC1 and PC2 accounted for 67.40% and 20.56% of the variation, respectively (total 87.96%). According to Yan and Kang (2003), longer environmental vectors indicate stronger discriminating power. In this study, Gwebi 2021 and Gwebi 2022 showed the longest vectors and thus were the most effective sites for distinguishing genotype performance.

Genotypes close to the biplot origin (e.g., G3, G5, and G17) exhibited small interaction scores, indicating high stability and broad adaptation. In contrast, genotypes positioned farther away (e.g., G14, G15, G18, G19) had large IPCA scores, reflecting strong GE interaction and environment‐specific responses. Although PC1 and PC2 together captured 87.96% of the total variation in β‐carotene, no environment aligned with a vertex genotype. G14 and G17 formed vertices without representing any environment, while G20 performed well overall but was not a vertex genotype.

**FIGURE 2:**
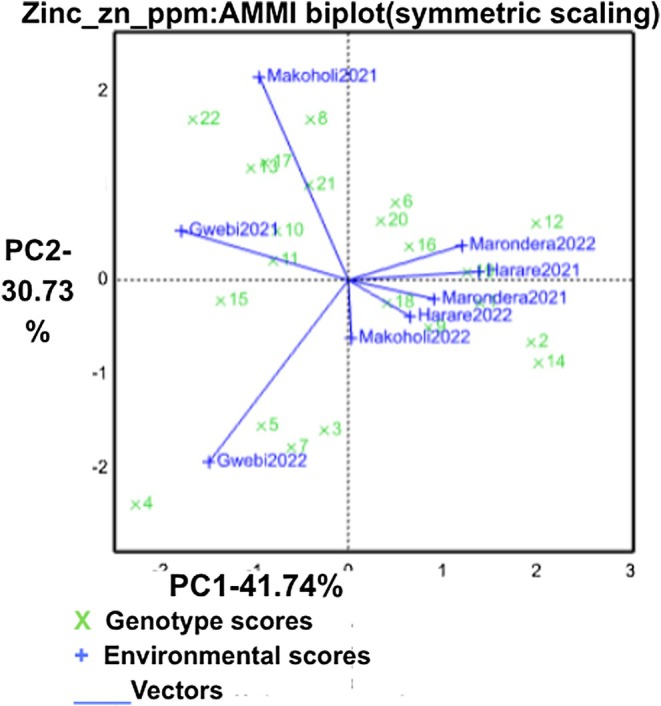
GGE‐biplot showing interrelationship among environments and discriminating ability of the environments for Zinc.

The AMMI biplot for Zn (Figure 2) explained 72.47% of GE interaction variation (PC1 = 41.74%, PC2 = 30.73%). Genotypes such as G1, G11, and G21, located near the origin, showed minimal GE interaction and therefore consistent Zn concentration across sites. Genotypes positioned farther from the center, including G4, G5, G3, and G22, demonstrated strong GE interaction, indicating environment‐dependent performance. For example, G4 aligned with environments in the lower left quadrant, while G12 and G2 aligned with environments toward the right of the biplot, highlighting specific adaptation.

**FIGURE 3:**
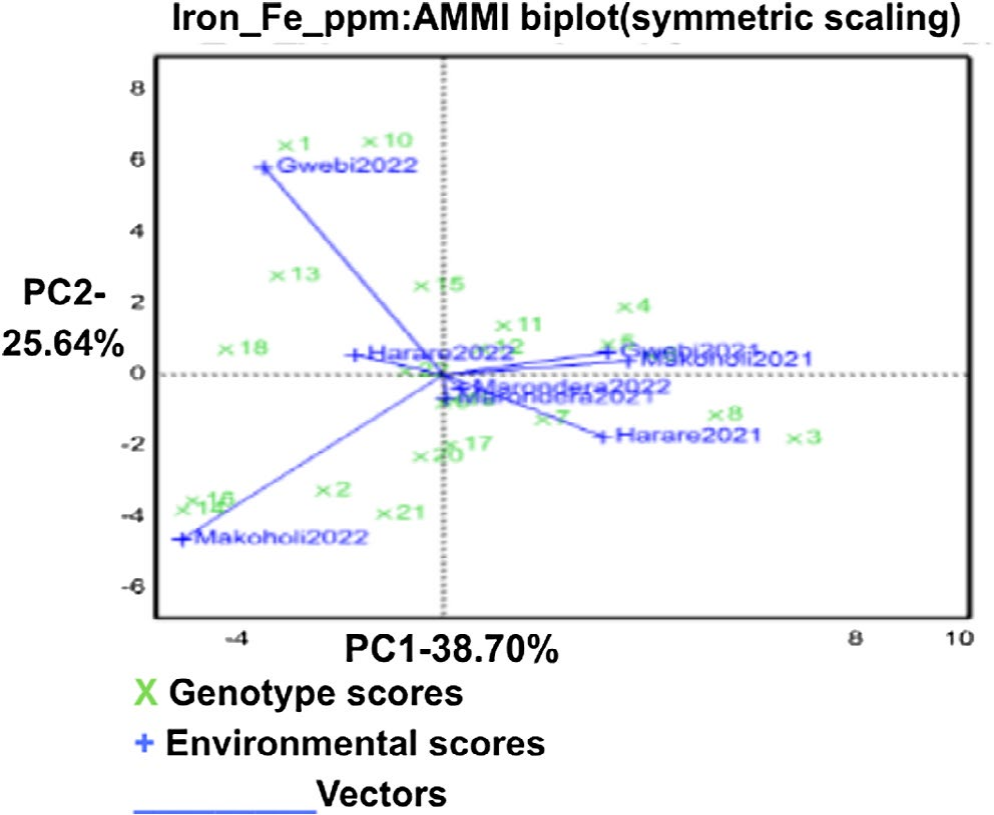
GGE‐biplot showing interrelationship among environments and discriminating ability of the environments for Iron.

For Fe concentration the GGE biplot (Figure 3), PC1 and PC2 explained 38.70% and 25.64% of GE interaction, respectively (total 64.34%). Genotypes located near the biplot origin (G11, G4, G5, G20, G17, G21, G2) showed low GE interaction and thus stable performance and wide adaptation. In contrast, genotypes positioned further from the origin displayed stronger environment specific responses: G10, G1, and G13 in the upper‐left quadrant showed affinity for environments in that direction, while G14, G3, G8, G12, and G22 aligned with environments on the right side of the plot, indicating more targeted adaptation for Fe accumulation.

## 4 Discussion

### 4.1 Variability and Genotype × Environment Interaction Effects in Root Yield and Micronutrient Concentrations

The significant genotype variation found in this study for β‐carotene, Fe, and Zn suggests that there is enough genetic variation for breeding and improving these traits. Crop improvement is hinged on the availability of genetic variation for selection and population development (Acquaah 2020). However, the significant genotype × environment interactions found could complicate the selection process. The GxE interaction effects show that genotype ranking for nutritional content changed between environments. This means that the selection of high‐performing genotypes in one environment may not correspond to another environment. Subsequently, selection should be conducted in individual locations, which increases breeding costs (Crossa et al. 2021). The interactions also offer opportunities for identifying widely adapted genotypes. Genotypes that are consistently ranked high in multiple environments are widely adapted and can be recommended for production across a wider network of environments.

In this study, the genotype‐by‐environment interaction (GEI) for root yield was minimal and not statistically significant, indicating that genotype rankings for root yield remained fairly consistent across different environments, even though root micronutrient levels varied. As noted by Welch and Graham (2004) and Beebe (2020), such results suggest that the genetic traits influencing root yield are relatively stable across various testing conditions, leading to consistent performance of genotypes. These findings imply that reliable assessment of genotypes for root yield can be achieved using fewer testing locations, which could help reduce the overall cost of field trials.

### 4.2 Yield Stability

As reported by Purchase et al. (2000), genotypes with lower AMMI Stability Values (ASV) are considered more stable across different environments. In this study, sweetpotato genotypes G3, G19, G16, G22, and G4 exhibited the lowest ASV scores for fresh root yield, indicating a high degree of yield stability across the evaluated environments.

### 4.3 GGE Biplot Analysis

The AMMI biplot for β‐carotene shows clear GE interaction, evidenced by the wide dispersion of genotypes along the IPCA axes. This confirms that β‐carotene accumulation is highly environment‐dependent, consistent with reports that micronutrient traits often display strong GE effects (Gauch Jr. 2013; Yan and Kang 2003). Genotypes G3, G5, and G17 cluster near the origin, indicating minimal interaction and broad stability, making them suitable for wide adaptation (Purchase et al. 2000; Kaya et al. 2002). In contrast, G14, G15, G18, and G19 lie farther from the origin, showing strong interaction and specific adaptation, performing best only in favorable environments (Gauch Jr. 2013; Yan and Tinker 2006).

For Fe content, the AMMI biplot similarly identifies genotypes with wide stability G11, G4, G5, G20, G17, and G21 based on their proximity to the origin (Gauch 2006). Genotypes positioned along distinct environmental vectors, such as G10 and G1 for Gwebi2022 and G14 and G16 for Makoholi2022, indicate targeted adaptation. The long vector for Gwebi2022 demonstrates strong discriminative ability, making it an efficient site for Fe‐based selection (Yan and Tinker 2006).

The Zn AMMI biplot also reveals substantial GE interaction, confirming the strong environmental influence on Zn accumulation (Gauch Jr. 2013). Genotypes G1, G11, and G21 appear near the origin and show broad stability, whereas G4, G5, G3, G12, and G2 exhibit specific adaptation to environments like Gwebi2022 or Marondera2022. Again, Gwebi2022 is highly discriminative for Zn, supporting its use as a key evaluation site. Overall, Zn results support a dual strategy: selecting stable genotypes for broad deployment and utilizing environment‐responsive genotypes for targeted improvement.

The findings of this study are consistent with previous reports on sweetpotato micronutrient variability. For example, recent studies have reported substantial variation in β‐carotene content among orange‐fleshed sweetpotato genotypes, highlighting the effectiveness of biofortification efforts (Naidoo et al. 2021; Mwanga et al. 2021). These studies reinforce the role of genotype and breeding programs in improving nutritional quality in sweetpotato.

The micronutrient ranges observed in this study are comparable to those reported in biofortified sweetpotato germplasm evaluations, suggesting that the evaluated materials fall within nutritionally relevant ranges (Low et al. 2007). However, compared to other studies, the relatively moderate Fe and Zn levels observed here may reflect soil variability and genotype‐specific uptake efficiency, emphasizing the need for integrating agronomic biofortification strategies.

## 5 Conclusions

For the majority of developing‐world communities, sweetpotato is an essential staple crop. The high yielding genotypes were G2, G19, G16, G22, and G3. The levels of β‐carotene were high in G19, G12, and G16. Different test sites had different ranks for the best genotypes in terms of iron, zinc, and beta carotene. G19 and G16 are recommended for promotion in areas where both yield and nutritional quality (particularly β‐carotene) are priorities, due to their consistent performance across traits. The polygon (“which‐won‐where”) view of the biplot helps identify mega‐environments and their winning genotypes, while the average environment coordination (AEC) method allows evaluation of both mean performance and stability. In this study, environments such as Gwebi 2021 and Gwebi 2022 exhibited long vectors, indicating strong discriminative ability and suitability for genotype evaluation. Genotypes located near the AEC abscissa with shorter projections were considered more stable, whereas those further away showed specific adaptation. These interpretations enhance the practical application of GGE results in breeding programs by enabling breeders to select both widely adapted and specifically adapted genotypes.

Further multi‐location trials should be conducted to confirm the stability of nutrient accumulation (especially Fe and Zn) across diverse agro‐ecologies. Incorporation of location‐specific soil and agronomic data is recommended to guide targeted variety deployment and fertilizer recommendations. Where resources allow, farmers and extension services should adopt simple tools like color charts or mobile‐based apps for rapid assessment of root color and potential provitamin A content in the field. Breeding programs should continue to focus on multi‐trait selection not only for yield but also for micronutrient density while validating trait stability under different environmental conditions. Finally, on‐farm participatory evaluations are recommended to assess acceptability, storage life, and cooking quality of the promising genotypes, ensuring their successful adoption by local communities.

## Funding

The authors have nothing to report.

## Conflicts of Interest

The authors declare no conflicts of interest.

## Supporting information


**Data S1:** Chizhande Data used.

## Data Availability

The datasets generated and analyzed during the current study are available in the Supporting Information accompanying this article.
